# Local but not systemic administration of mesenchymal stromal cells ameliorates fibrogenesis in regenerating livers

**DOI:** 10.1111/jcmm.14508

**Published:** 2019-06-27

**Authors:** Danny van der Helm, Marieke C. Barnhoorn, Eveline S. M. de Jonge‐Muller, Ilse Molendijk, Luuk J. A. C. Hawinkels, Minneke J. Coenraad, Bart van Hoek, Hein W. Verspaget

**Affiliations:** ^1^ Department of Gastroenterology and Hepatology Leiden University Medical Center Leiden The Netherlands

**Keywords:** CCl4, cirrhosis, fibroblasts, fibrogenesis, fibrosis, liver, mesenchymal stem cells, MSC, regeneration

## Abstract

Chronic liver injury leads to the accumulation of myofibroblasts resulting in increased collagen deposition and hepatic fibrogenesis. Treatments specifically targeting fibrogenesis are not yet available. Mesenchymal stromal cells (MSCs) are fibroblast‐like stromal (stem) cells, which stimulate tissue regeneration and modulate immune responses. In the present study we assessed whether liver fibrosis and cirrhosis can be reversed by treatment with MSCs or fibroblasts concomitant to partial hepatectomy (pHx)‐induced liver regeneration. After carbon tetrachloride‐induced fibrosis and cirrhosis, mice underwent a pHx and received either systemically or locally MSCs in one of the two remaining fibrotic/cirrhotic liver lobes. Eight days after treatment, liver fibrogenesis was evaluated by Sirius‐red staining for collagen deposition. A significant reduction of collagen content in the locally treated lobes of the regenerated fibrotic and cirrhotic livers was observed in mice that received high dose MSCs. In the non‐MSC‐treated counterpart liver lobes no changes in collagen deposition were observed. Local fibroblast administration or intravenous administration of MSCs did not ameliorate fibrosis. To conclude, local administration of MSCs after pHx, in contrast to fibroblasts, results in a dose‐dependent on‐site reduction of collagen deposition in mouse models for liver fibrosis and cirrhosis.

## 1 INTRODUCTION

The liver is an organ with multiple important roles in detoxification, metabolism, immune defence and homoeostasis. External factors like viral hepatitis infection, chronic alcohol abuse, non‐alcoholic steatohepatitis and metabolic‐ and cholestatic disease can cause chronic liver damage, leading to hepatic fibrogenesis. This process can eventually result in end‐stage liver cirrhosis and liver failure. Fibrogenesis is the result of a complex cellular interplay between apoptotic hepatocytes, inflammatory cells, biliary epithelial cells, Kupffer cells and stellate cells.^1^, ^2^, ^3^ In this process, apoptotic hepatocytes are thought to induce the activation and increased proliferation of stellate cells and their subsequent differentiation into myofibroblast. These myofibroblasts play a central role in liver fibrosis and are responsible for the characteristic production of excessive amounts of extracellular matrix (ECM).^1^, ^2^, ^3^

To date, curing of the underlying disease is the only treatment for fibrosis. For instance, the case of sustained viral response to treatment for hepatitis C‐virus, can lead to reversal of fibrogenesis.^3^, ^4^ Therapeutic drugs or interventions which can specifically target fibrosis or the process of fibrogenesis are not yet available. Orthotopic liver transplantation (OLT) is the only available treatment for end‐stage liver cirrhosis.^5^, ^6^ As OLT is a major surgical intervention and medical undertaking with inherent complications and risks and is dependent on patient condition and donor availability, alternative strategies including hepatocyte transplantation and potential anti‐fibrotic drugs are being explored.^7^, ^8^, ^9^

Mesenchymal stromal cells (MSCs) are fibroblast‐like multipotent stromal (stem) cells which can be isolated from bone marrow, adipose tissue and umbilical cord. MSCs expand easily in vitro and are not rejected upon transplantation.^10^, ^11^, ^12^ Furthermore, MSCs are able to modulate inflammatory responses, and the repair and regeneration of damaged tissues. These characteristics make them attractive candidates for prevention and treatment of liver fibrosis where these specific processes need to be restored in order to reverse fibrogenesis.^11^, ^12^, ^13^ Currently, MSCs have been tested in clinical trials with promising, but also sometimes ineffective, results regarding the reversal of fibrosis, cirrhosis and end‐stage liver disease.^14^, ^15^, ^16^ Several working mechanisms of MSCs have been proposed, including their ability to differentiate into hepatocytes, to stimulate the protection and survival of liver resident cells, to inhibit the activation of stellate cells and to silence the myofibroblasts.^11^, ^17^, ^18^, ^19^, ^20^ However, the exact mechanisms of action of MSCs in reducing liver fibrosis are still unknown.

Previous studies in mice and zebrafish embryos showed that MSCs are able to prevent chemically induced hepatic fibrosis when administered simultaneously with the causative agent.^21^, ^22^, ^23^, ^24^ Some other in vivo studies showed that MSCs are also effective to treat carbon tetrachloride (CCL4) established fibrosis.^15^ MSCs are fibroblast‐like cells with several functions and characteristics similar to fibroblasts. Some studies claim that fibroblasts, like MSCs, have the same capacity to suppress the immune system and that they also play a role in tissue repair.^25^, ^26^, ^27^ However, no studies have been reported comparing both cell types in relation to liver fibrosis.

The liver has a high regenerative capacity upon tissue damage, for example after resection or because of hepatotoxic substances.^4^, ^17^, ^28^, ^29^ None of the published studies combined MSC therapy with this regenerative capacity of the liver. Therefore, we explored if the combination of the intrinsic regeneration capacities of the liver and the pro‐regenerative and anti‐inflammatory capacities of MSC therapy could ameliorate liver fibrogenesis.

As also mentioned by Hu et al, results from literature are difficult to compare as there are multiple differences in study design such as disease aetiology, disease stage and the administration route and dosage and source of MSCs, which possibly all could affect the outcome of these studies.^30^ Therefore, in the present study, for the first time, the effects of different administration routes of MSCs (local vs iv), different disease stages (fibrosis vs cirrhosis) and different MSC dosages were evaluated and compared in the same study. Furthermore, we compared the therapeutic potential of MSCs and fibroblasts in a novel treatment strategy where mice with CCL4‐induced fibrosis and cirrhosis underwent a partial hepatectomy (pHx), as regeneration stimulus, and received concomitant cell therapy. We suggested that specifically administration of MSCs in regenerating fibrotic and cirrhotic livers would be able to ameliorate fibrogenesis.

## 2 MATERIAL AND METHODS

### 2.1 MSC and fibroblast isolation culturing and characterization

Bone marrow‐derived MSCs and liver‐derived fibroblasts were isolated from 10‐week‐old actin‐GFP C57Bl/6Jico mice obtained from an LUMC breeding population.^31^ In short, mice were killed by cervical dislocation and the liver, femur, tibia and humerus were collected. For MSC isolation, bones were cleaned from tissue and flushed with RPMI medium supplemented with 10% foetal calf serum (FCS; Gibco, Paisley, UK), 3 mmol/L l‐glutamine (Invitrogen Corp., Paisley, UK), penicillin/streptomycin (P/S; Invitrogen Corp., Paisley, UK) and 2% Heparin (Pharmacy LUMC, Leiden, The Netherlands). Collected cells were cultured in αMEM culture medium (Lonza, BE12‐169F) supplemented with 10% FCS, 3 mmol/L l‐glutamine and P/S (complete culture medium). After 24, 48 and 72 hours non‐adhering cells and cell debris were removed. To isolate fibroblasts, livers were cut in small parts and incubated with Liberase^TM^ LT (Roche, Basel, Switzerland) for 30 minutes at 37°C. Next, the cell suspension was washed and subsequently cultured in DMEM/F12 culture media supplemented with 10% foetal calf serum, P/S, Hepes buffer solution and gentamicin (both Gibco). Cultured cells were used until passage 8‐10. Cells were monthly tested for mycoplasma contamination. Isolated cells were characterized by the expression of membrane markers and their ability to differentiate into osteoblasts and adipocytes (Supplementary Material).

### 2.2 Fibrotic and cirrhotic mouse model

All mice received food and water ad libitum and were housed in individually ventilated cages. All animal experiments were approved by the animal ethics committee of the Leiden University Medical Center. For the fibrotic and cirrhotic models 6‐week‐old male C57Bl/6Jico mice (Charles River Laboratories, The Netherlands) were used. For fibrosis induction, mice received three intraperitoneal (ip) CCL4 injections (100 µg/kg body weight) per week for 6 weeks (Sigma‐Aldrich Chemie BV, Zwijndrecht, The Netherlands) (Figure S1A). For induction of cirrhosis, mice were treated for 11 weeks with two initial doses of 200 µg/kg body weight CCL4, followed by a twice weekly 150 µg/kg body weight CCL4 ip injection for 10 weeks (Figure S1B). All CCL4 injections were diluted to an injection volume of 50 µL with mineral oil (Sigma‐Aldrich Chemie BV, Zwijndrecht, The Netherlands). After 6 weeks (fibrosis) or 11 weeks (cirrhosis) a pHx was performed, as described previously by Anderson and Higgins.^32^ In short, animals were anaesthetized and the two median and the left lateral lobes were ligated and removed (50%‐70% of the liver, Figure S1C). Next, mice were randomly divided into four groups and were locally treated with vehicle (saline), 1 x 10^6^ or 2 x 10^6^ MSCs or 2 x 10^6^ fibroblasts divided over five spots in one of the remaining liver lobes (lobe 5, Figure S1C). The tail vein administration group received 1 x 10^6^ MSCs one day before and one day after pHx (2 x 10^6^ MSCs in total). This group received two injections of 1 x 10^6^ MSCs as higher systemic dosages led to too high numbers of animal loss. Two groups with fibrosis did not undergo pHx and received no further treatment or received local administration of 2 x 10^6^ MSCs. Eight days after pHx, the mice were killed and livers were resected, weighted and fixated for paraffin embedding and stored for protein isolation.

### 2.3 Transaminase levels

Blood from the tail vein was collected before the start of CCL4 administration, the day before pHx and 8 days after pHx (Figure S1A,B). Alanine aminotransferase (ALT) and aspartate aminotransferase (AST) serum levels were measured with Reflotron equipment (Roche diagnostics GmbH, Mannheim, Germany) according to the manufacturers’ instructions.

### 2.4 TNF‐α measurement

Liver homogenates were made with a Potter‐Elvehjem glass homogenizer at 4°C in Greenberger lysis buffer. Homogenates were centrifuged (15 minutes, 11 000 *g*, 4°C) and stored at −80°C. BCA Protein Assay Kit (Thermo Scientific Pierce, Etten‐Leur, The Netherlands) was used to measure total protein content in the homogenates. TNF‐α protein levels were measured using the Cytometric Bead Array System (BD Biosciences, San Diego, CA, USA) according to the manufacturer's instructions. Data analysis was performed with FlowJow software. TNF‐α levels were corrected for the total amount of extracted protein.

### 2.5 Histological examination

Paraffin sections of 4 µm were cut, rehydrated and stained for 90 minutes with 1 g/L Sirius‐red (Klinipath Sirius F3B) in picric acid (Klinipath) to visualize collagen deposition. Next, slides were cleared with 0.01 mol/L HCl, washed, dehydrated and mounted with Entellan (Merck KGaA, Darmstadt, Germany). Collagen deposition was quantified by taking 5‐8 random images (10× magnifications) of Sirius‐red stained sections with fixed microscopy settings. Subsequently, the amount of staining was quantified with ImageJ (ImageJ 1.47v, National Institutes of Health, USA) and the reduction of collagen content in the liver, relative to the resected pHx tissue, calculated.

Lobuli closure was used as a second measure of fibrosis and cirrhosis and was performed blindly by two independent observers. In short, the liver is organized in lobuli with a hexagonal figure consisting of six portal triads with one central vein in the middle. In a fibrotic liver the excessive collagen is secreted into the space between the portal triads and forms septa. When fibrogenesis is sustained for a longer period, the septa will grow and eventually bridge the space between the portal triads, which correlates with a more severe fibrosis and finally cirrhosis (Figure S2 for lobuli closure scoring method).

### 2.6 Immunohistochemistry

For the staining of green fluorescent protein (GFP) and α‐smooth muscle actin (α‐SMA), paraffin‐embedded tissue sections were rehydrated, and endogenous peroxidases were blocked with 0.3% H_2_O_2_/methanol followed by a 10‐minutes boiling citrate antigen retrieval (pH6). Next, sections were blocked with 2% horse serum in PBS containing 0.1% Triton X‐100 and 1% bovine serum albumin. Primary antibodies for GFP (Rockland, cat 600101215), and α‐SMA (Progen, Heidelberg, Germany, cat 61001) were incubated overnight. The next day, slides were incubated with a peroxidase‐labelled polymer (EnVision+, Dako Netherlands BV, Heverlee, Belgium) after which the staining was visualized with 3,3’‐diaminobenzidine (DAB Fast Tablet, Sigma‐Aldrich, St. Louis, MO). After nuclear counterstaining with haematoxylin, slides were dehydrated and mounted with Entellan.

### 2.7 Statistical Analysis

Student's *t* test was used to compare two groups. For comparison of three or more groups one‐way ANOVA was used followed by Dunnett's multiple comparison tests. The results are presented as mean ± standard error of the mean (SEM). Statistical tests were performed with GraphPad Prism software (GraphPad Software, version 5.01, San Diego, CA). *P* < 0.05 were considered as statistically significant.

## 3 RESULTS

### 3.1 Characterization of MSCs and fibroblasts

Flow cytometry revealed that the MSCs were positive for the MSC membrane markers: CD29, CD105, CD106, SCA‐1, CD44 and negative for haematopoietic marker CD45 and endothelial marker CD31 (Figure S3A). Fibroblasts showed a similar expression pattern, except for SCA‐1 that was not detected in the fibroblasts (Figure S3B). Further characterization revealed that MSCs showed adipogenic and osteoblastic differentiation potential (Figure S4). Fibroblasts were able to differentiate into adipocytes but did not differentiate into osteoblasts (Figure S4). Taken together these data indicate that the MSCs fulfilled the criteria for MSCs, whereas the fibroblasts did not.

### 3.2 CCL4‐induced liver fibrosis and cirrhosis in mice

**Figure 1:**
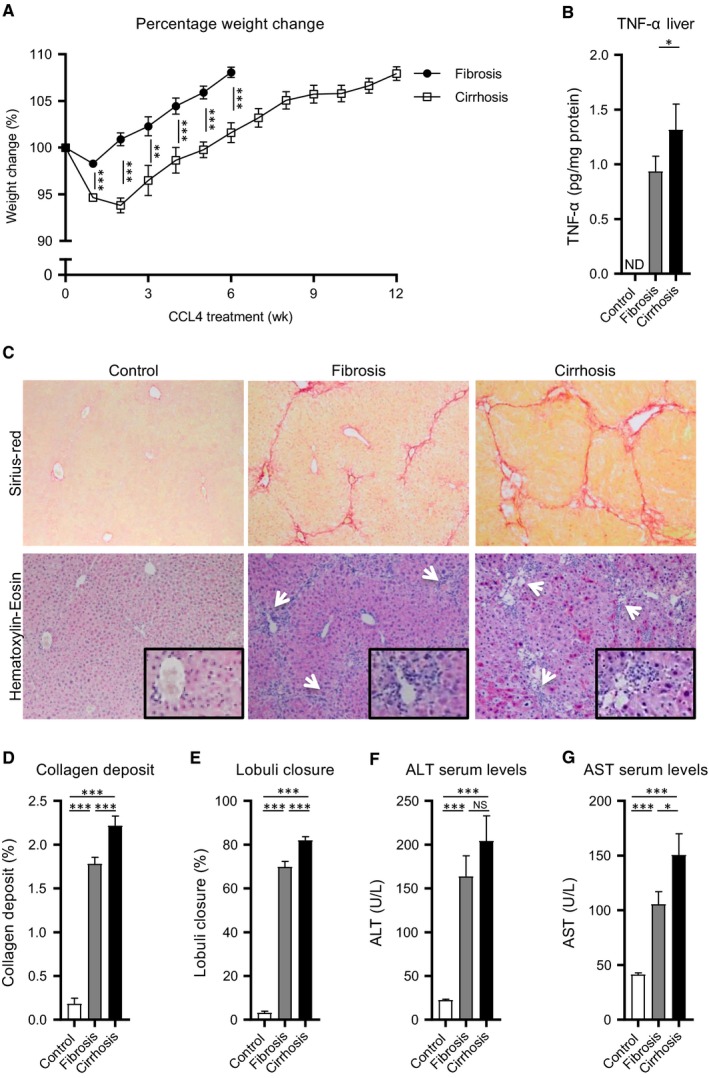
CCL4‐induced liver fibrosis and cirrhosis in mice. A, Normalized body weight during the induction of fibrosis and cirrhosis (N = 25). B, TNF‐α levels in healthy and resected liver tissues (N = 8). C, Sirius‐red and Haematoxylin‐eosin stained healthy, fibrotic and cirrhotic liver tissue (20x magnifications, inserts 40x magnifications). D, Quantified collagen deposit of Sirius‐red stained sections (N = 25). E, Estimated lobuli closure of Sirius‐red stained sections (N = 25). F and G, ALT and AST serum levels of healthy (N = 35), fibrotic (N = 30) and cirrhotic animals (N = 38). Data are expressed as mean ± SEM. *P ≤ 0.05, **P ≤ 0.01, ***P ≤ 0.001. ALT, alanine aminotransferase; AST, aspartate aminotransferase; CCL4, carbon tetrachloride; ND, not detectable; NS, not significant

For the present study, mouse models representing fibrotic and cirrhotic disease stage were generated by CCL4 administration. During the first 6 weeks of CCL4 treatment, cirrhotic mice had a significantly lower body weight compared to the fibrotic mice (Figure 1A). After the induction of fibrosis and cirrhosis three liver lobes were resected and used to evaluate the severity of fibrogenesis. TNF‐α levels in the liver, as a marker for inflammation, were significantly higher in mice with cirrhosis compared to mice with fibrosis or a healthy liver (Figure 1B). Sirius‐red staining of the resected fibrotic and cirrhotic liver tissue showed significantly increasing levels of collagen deposition and lobuli closure indicative for progressive fibrogenesis in these groups respectively (Figure 1C‐E). Furthermore, morphological cell analysis of H&E staining of the tissues illustrated increased numbers of myofibroblasts and infiltrating lymphocytes in the septa between the portal triads in these respective groups (Figure 1C, white arrows). The day before pHx, aminotransferase levels were measured. Alanine transaminase and aspartate transaminase serum levels were increased upon CCL4 injury and reached higher levels in cirrhotic mice, compared to fibrotic or healthy mice respectively (Figure 1F,G). Because of the toxicity and prolonged exposure of CCL4, more mice had to be prematurely killed during the induction of cirrhosis (40%) compared to the induction of fibrosis (17%). In combination, these results indicate that mice in the cirrhosis group had more severe liver damage at time of pHx, compared to the mice in the fibrosis group. These observations represent the starting point for the MSCs treatment experiments.

### 3.3 Systemically administrated MSCs did not further improve the pHx initiated reversal of fibrosis

**Figure 2:**
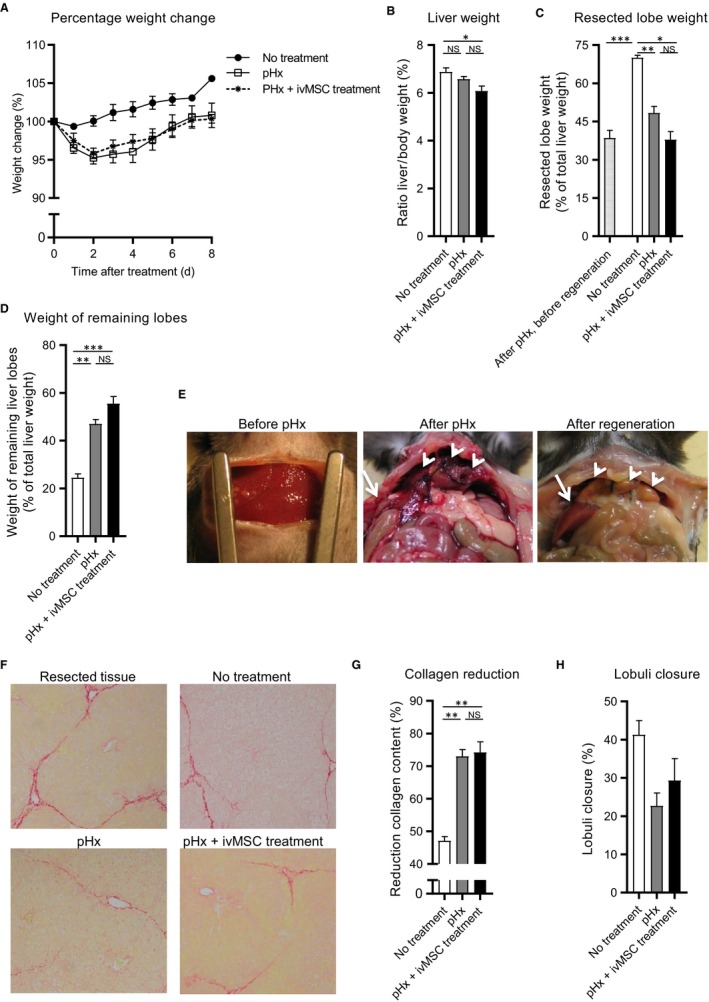
Systemically administrated MSCs did not further improve the pHx initiated reversal of fibrosis. Mice with liver fibrosis received no treatment, pHx or pHx + ivMSC (N = 6/9 per group). A, Normalized body weight during regeneration. Relative weights of (B) total liver, (C) remnant parts of the resected lobes and (D) remaining lobes after regeneration. E, Pictures of the liver before pHx, after pHx and after regeneration. Remnant and remaining lobes are indicated with white arrow heads and white arrows respectively. F, Sirius‐red stained liver tissue (20x magnifications) (G) Reduction of Sirius‐red staining relative to resected tissue. H, Estimated lobuli closure. Data are expressed as mean ± SEM. *P ≤ 0.05, **P ≤ 0.01, ***P ≤ 0.001. MSCs, mesenchymal stromal cells; pHx, partial hepatectomy; NS, not significant

To address the potential of MSCs to reverse liver fibrosis, MSCs were systemically administered by tail vein injection. One group of CCL4‐treated mice received MSCs iv One day prior to and 1 day after pHx (pHx + ivMSC), the other group had only a pHx and the last group received no treatment (natural recovery after CCL4). In the first 2 days after surgery, the mice in the pHx and pHx + ivMSC group seemed to lose slightly but not significantly more weight as compared to the group that received no treatment (Figure 2A). After 8 days, no differences in body weights were observed. After regeneration, livers were collected, weighted and stained for collagen by Sirius‐red staining. The pHx + ivMSC‐treated mice had relatively smaller livers compared to the pHx group and to the no treatment group (Figure 2B). In order to compensate for the resected liver volume, the non‐resected, remaining liver lobes had grown (lobes 4 and 5 in Figure S1C, Figure 2C‐E, white arrows). After regeneration, no differences in relative weights of these remaining liver lobes between the pHx and pHx + ivMSC groups were observed (Figure 2D). The remnant parts of the resected liver lobes remained small and did not regenerate (white arrowheads, Figure 2C,E).

The Sirius‐red staining showed that the pHx significantly reduced the total collagen deposition, independently of the iv administrated MSCs (72% and 73% reduction, Figure 2F,G). Scoring based on lobuli closure resulted in a corresponding trend towards less closure of the pHx (23%) and pHx + ivMSCs (29%) group compared to the no treatment (41%) group (Figure 2H).

Altogether, these data showed that only a pHx already leads to a considerable reduction in the collagen content of the regenerating livers and that the ivMSC treatment does not have an additional effect on this collagen reduction.

### 3.4 Local administration of MSCs during pHx reduces collagen content of regenerating livers in a fibrotic mouse model, whereas fibroblast administration does not

**Figure 3:**
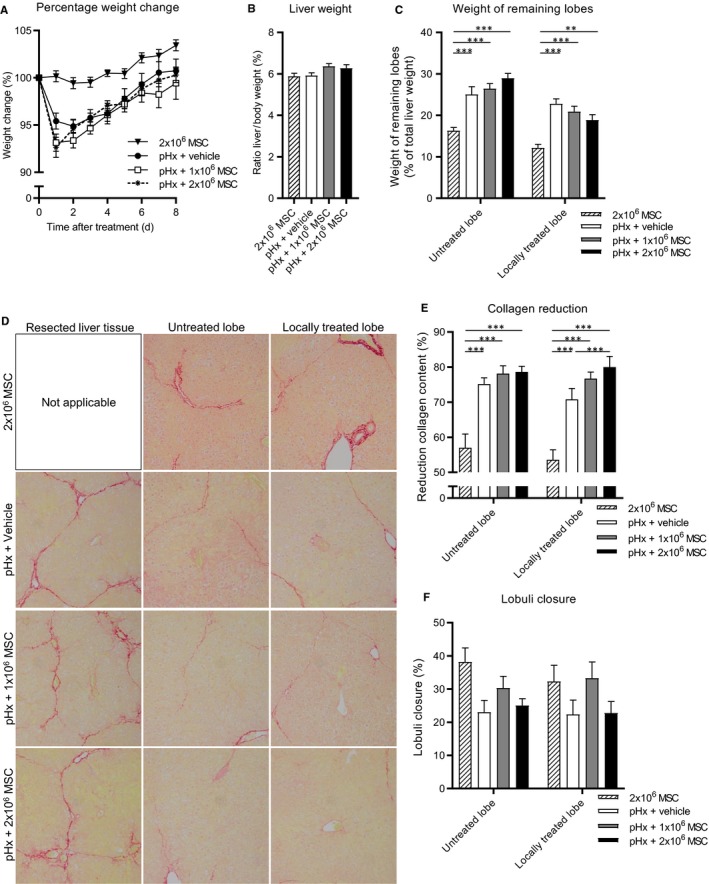
Local administration of MSCs during partial hepatectomy reduces collagen content of regenerating livers in a fibrotic mouse model. After induction of fibrosis mice received local 2 x 10^6^ MSC treatment or underwent pHx and local treatment with vehicle, 1 x 10^6^ or 2 x 10^6^ MSCs (N = 15 per group). A, Normalized body weight during regeneration. B, Normalized liver weight and (C) relative weights of treated and untreated lobes as percentage liver after regeneration. D, Sirius‐red stained sections of resected, untreated and treated remaining liver lobe tissue of the different treatment groups (20x magnifications). E, Reduction of Sirius‐red staining relative to resected tissue. F, Estimated lobuli closure. Data are expressed as mean ± SEM. **P ≤ 0.01, ***P ≤ 0.001. MSCs, mesenchymal stromal cells; pHx, partial hepatectomy

Next, we assessed whether local MSC therapy could enhance the effect of the pHx‐induced collagen reduction. Therefore, MSCs were locally injected underneath the liver capsule in one of the remaining lobes after pHx (Figure S1C). As a control, one group of mice received local MSC therapy without pHx. After pHx, the 1 x 10^6^ and 2 x 10^6^ MSC groups lost slightly but not significantly more weight compared to the vehicle control group (Figure 3A). Mice that received only local MSC therapy did not lose body weight after treatment. At the end of the experiment no differences in body weight and relative liver weight were observed and livers were fully regenerated in all groups (Figure 3A‐C). The groups that received pHx showed bigger liver lobes compared to the corresponding lobes of the group that only received local MSC treatment (Figure 3C). In addition, no differences in serological ALT and AST levels were observed (Figure S5A,B).

Sirius‐red stained liver tissue showed that 2 x 10^6^ MSC treatment without pHx had led to more reduction of collagen content compared to no treatment but less reduction compared to mice that received pHx + vehicle (Figure 2G and 3E). Furthermore, the reduction of collagen deposition in mice that received pHx and MSCs was related to the number of administered MSCs. Collagen reduction was higher in pHx + 2 x 10^6^ MSC (80%)‐treated animals compared to the pHx + 1 x 10^6^ MSC (77%) and pHx + vehicle (71%) group respectively (Figure 3D,E). No differences in the reduction of collagen content in the non‐treated liver lobes between the same three groups of mice were observed, suggesting an on‐site effect of MSCs in this model. When pHx + vehicle and pHx + ivMSC treatment were compared to the pHx + local administration of 2 x 10^6^ MSCs, we concluded that local administration of MSCs enhanced the pHx‐induced reduction of collagen deposition (72%, 73% vs 80%, Figures 2G and 3E). No significant difference in lobuli closure was observed (Figure 3F).

**Figure 4:**
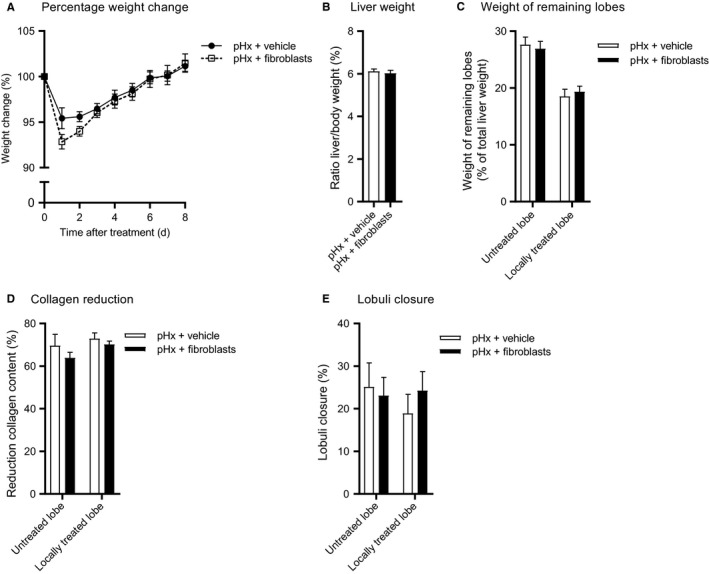
Fibroblasts are unable to resolve fibrosis in regeneration mouse livers. After fibrosis induction, mice underwent pHx and received local administration of vehicle or 2 x 10^6^ fibroblasts (N = 9 group size). A, Normalized bodyweight during regeneration (B) Normalized liver weight and (C) relative treated and untreated lobe weights as percentage liver after regeneration (D) Reduction of Sirius‐red staining relative to resected tissue. E, Estimated lobuli closure. Data are expressed as mean ± SEM. pHx, partial hepatectomy; SEM, standard error of the mean

Next, the ability of liver fibroblasts to modulate the regenerative process was examined. In this experiment, CCL4‐treated mice underwent a pHx with or without local fibroblast treatment. At the end of the experiment no differences in body weight and relative liver weight were observed in mice which received fibroblasts compared to control mice which received vehicle (Figure 4A‐C). Also Sirius‐red staining in these fibroblast experiments did not show differences in collagen reduction or lobuli closure (Figure 4D,E). Overall, these results indicate that local injection with MSCs, in contrast to local injection with fibroblasts, seems to enhance the effect on collagen reduction of the pHx initiated liver regeneration.

### 3.5 Local MSC treatment reduced the amount of collagen deposition in a mouse model for liver cirrhosis

**Figure 5:**
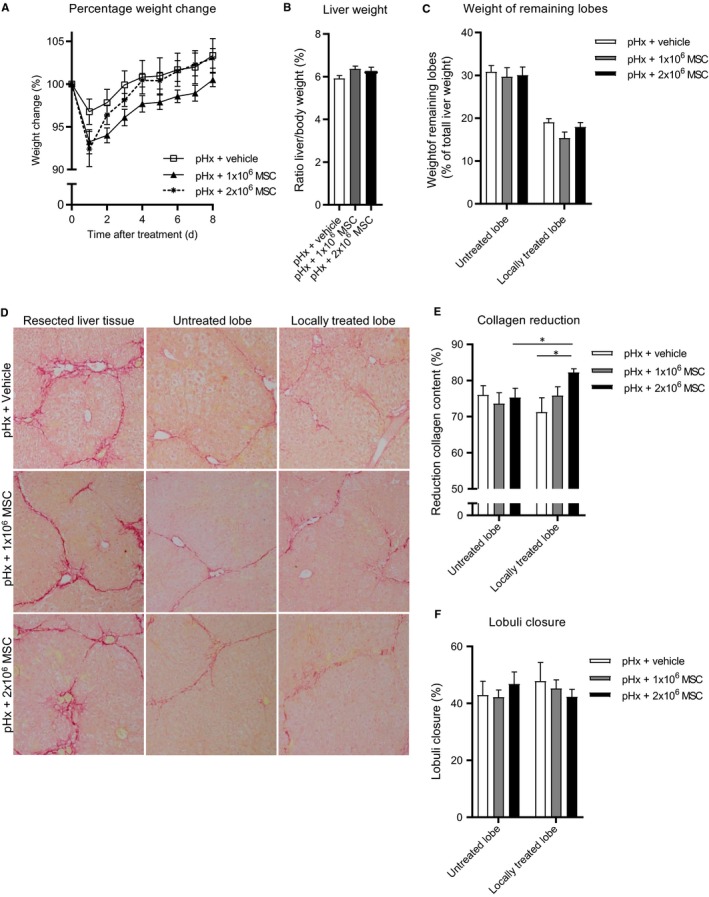
MSCs significantly decrease collagen deposition in a mouse model for liver cirrhosis. Mice with liver cirrhosis were treated by pHx and local administration of vehicle, or 1 x 10^6^ or 2 x 10^6^ MSCs (N = 8/10 group size). A, Normalized bodyweight during regeneration (B) Normalized liver weight and (C) relative treated and untreated lobe weights as percentage liver after regeneration (D) Sirius‐red stained sections of resected, untreated and treated remaining liver lobe tissue of the different treatment groups (20x magnifications). E, Reduction of Sirius‐red staining relative to resected tissue. F, Estimated lobuli closure. Data are expressed as mean ± SEM. *P ≤ 0.05. MSCs, mesenchymal stromal cells; pHx, partial hepatectomy

Subsequently, we evaluated if the observed therapeutic effect could also be reached in a more severe disease stage of fibrosis, that is, liver cirrhosis. After regeneration, slightly lower body weight was observed in the 1 x 10^6^ MSC group compared to the other groups (Figure 5A). After killing, livers and the individually separated liver lobes showed no differences in relative weight between the different treatment groups (Figure 5B,C). Furthermore, TNF‐α expression levels in the liver were below the detection limits in all samples (data not shown) and ALT and AST serum levels reached healthy baseline levels in all treatment groups (Figure S5C,D).

Sirius‐red stained tissue sections showed a significant relative reduction of collagen content in the locally treated liver lobe of the 2 x 10^6^ MSC treatment group (82%) compared to pHx + vehicle control group (71%). The 1 x 10^6^ MSC group reached an intermediate reduction of collagen deposition (76%, Figure 5D,E). More collagen reduction in the locally treated lobe (82%) vs the untreated counterpart (75%) in 2 x 10^6^ MSC group was observed (Figure 5E). The untreated liver lobes showed no differences between the different treatment groups, again indicating a local effect of the MSCs. Furthermore, lobuli closure showed a trend towards less closure in the 2 x 10^6^ MSC group, but this did not reach statistical significance. Also, no differences in lobuli closure between the untreated counterparts were observed further suggesting the importance of local MSC treatment (Figure 5F).

**Figure 6:**
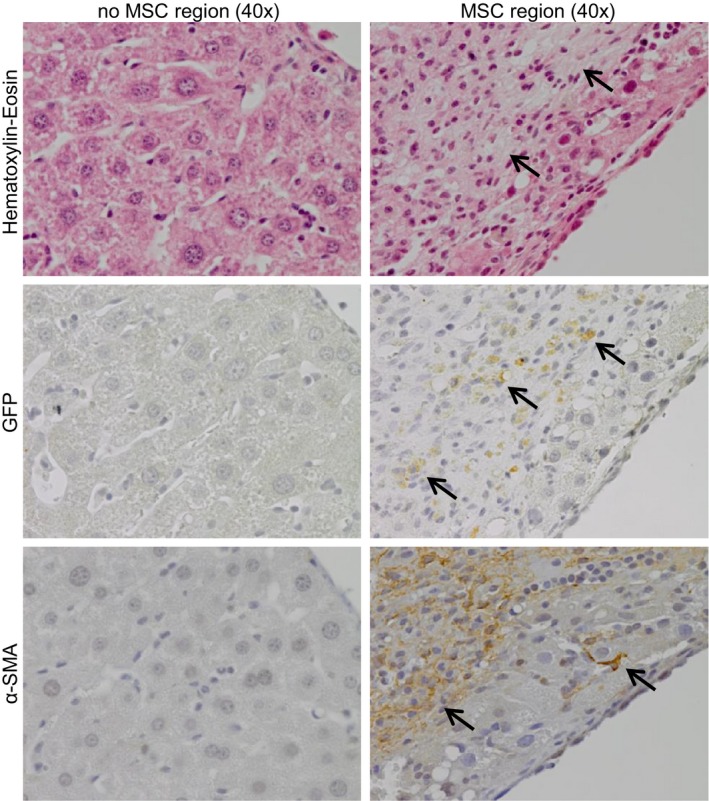
MSCs are traced in special organized regions. MSC regions and normal regions in regenerated liver tissue of cirrhotic mice treated with pHx + 2 x 10^6^ MSC stained for Haematoxylin‐eosin, GFP and α‐SMA (40x magnifications, MSCs are indicated by the black arrows). GFP, green fluorescent protein; α‐SMA, smooth muscle actin; MSCs, mesenchymal stromal cells; pHx, partial hepatectomy

At the end of the experiment, locally administered GFP‐expressing MSCs were traced at the injection site. Haematoxylin and eosin staining of regenerated liver tissue shows the well organized liver structure with hepatocyte plates. MSC regions were characterized by less well‐organized regions with few to no hepatocytes and multiple elongated, GFP‐ and α‐SMA‐positive cells, all indicative for MSCs (Figure 6, black arrows). MSCs were not observed outside these regions indicating that MSCs exert their anti‐fibrotic or pro‐regenerative effects from the injection site and do not migrate through the tissue. Altogether these results indicate an on‐site dose‐dependent effect of locally administered MSCs on collagen reduction in regenerating cirrhotic livers.

## 4 DISCUSSION

In the present observational study, the therapeutic efficacy in counteracting liver fibrogenesis by MSC or fibroblast therapy was tested in regenerating livers of mice with fibrosis or cirrhosis. Different dosages and administration routes of MSCs were evaluated to find the optimal therapy. Our data showed that local MSC treatment in combination with a pHx, as regeneration stimulus, dose dependently reduces collagen content in both a fibrotic and in a cirrhotic mouse model, while local administration of liver fibroblasts and systemic intravenous MSC administration had no effect. The locally administered MSCs were traced at the injection site from where they are thought to exert their function and locally reduce the collagen content in the regenerating livers.

Various studies used the CCL4‐based mouse models to evaluate potential therapeutic interventions, but they differ in the dose, frequency and duration of CCL4 administration, leading to differences in illness between studies and sometimes opposing results.^33^ In the present study, we established, described and compared chronic CCL4‐induced mouse models for liver fibrosis and cirrhosis in detail. At time of pHx, cirrhotic mice as compared to fibrotic mice had more liver damage based on lobuli closure, collagen deposit, aminotransferase levels and TNF‐α expression, indicating a more severe disease stage.

Several studies previously evaluated the ability of MSCs as potential treatment in fibrotic and cirrhotic animal models.^15^, ^20^, ^23^, ^34^ In line with these earlier studies, our results showed increased reduction of collagen levels upon MSC treatment. However, none of these previous studies included the regenerative response initiated by a pHx. In the present study, we aimed to enlarge the effect of MSC treatment by stimulating liver regeneration by performing a pHx. Our results showed that a pHx as such already leads to a reduction of collagen content and improve the effect of local MSC treatment and vice versa.

Beneficial characteristics of MSCs for reversing fibrosis and improving liver function include their ability to differentiate into hepatocytes, to stimulate proliferation and survival of resident liver cells, their immunosuppresive capicity and their ability to silence the collagen‐producing myofibroblasts.^11^, ^17^, ^18^, ^20^, ^34^, ^35^, ^36^ The precise working mechanisms are still unknown but probably are because of the combined action of these characteristics. In our study, an enhanced liver regeneration based on relative liver weights, in the mice treated with local MSC therapy, was not observed. These results indicate that the observed effect of local MSC treatment on relative collagen content is because of collagen reduction and not only to the regeneration of resident liver cells. Other studies have shown that the enhanced regeneration owing to MSC treatment could be observed at day 3.^17^ We examined the livers at day 8, and one could argue that this might be too late to find differences in liver weights as all livers were already fully regenerated at this time‐point.

In the present study, we did not find beneficial effects of iv adminstered MSCs. MSCs can easily get trapped in the lungs, which leads to fewer cells homing to the liver.^18^ This might be a possible explanation for the absence of an effect of iv administered MSCs. In contrast to iv MSC administration, local MSC administration in the liver during pHx did lead to more pronounced reduction of collagen deposition. Locally MSC‐treated lobes, when compared to the untreated counterparts, show a beneficial on‐site effect of MSCs, whereas no remote effect of MSCs was observed in the untreated counterparts. To our knowledge this is the first study describing this on‐site effect and could also further explain why the iv MSC treatment was ineffective. Particularly in the cirrhotic model the locally treated liver lobes of the pHx + 2 x 10^6^ MSC group reached significantly more reduction in collagen content compared to the untreated counterparts, underlining the importance of the local administration of MSCs. Thus, in general, a dose‐response effect between untreated, pHx, pHx + 1 x 10^6^ and pHx + 2 x 10^6^ MSCs was observed in the fibrotic and cirrhotic models. Furthermore, as described in previous studies, we observed that local MSC treatment leads to more reduction of collagen but in addition we showed that this effect can be improved by initiating a regenerative response by pHx.

The present study also compared MSCs and fibroblasts in their ability to resolve fibrosis. Studies by Haniffa et al showed that MSCs are fibroblast‐like cells with similar functions in immunosuppression and tissue repair.^25^ These studies, however, are not related to liver disease and focussed on basic mechanistic in vitro studies.^25^, ^26^, ^37^ We showed that MSCs and fibroblasts similarly express several membrane markers and both have adipogenic differentiation ability. In addition we found that MSCs, in contrast to fibroblasts, are positive for SCA‐1 and are able to differentiate into osteoblasts. These differences are also described by Cakiroglu et al who also demonstrate that fibroblasts are negative for SCA‐1.^38^ Furthermore, the present study revealed that MSCs but not the fibroblasts were able to reverse fibrogenesis in regenerating livers. These observations illustrate the unique phenotypical and functional features of MSCs. Fibroblasts may be considered as myofibroblast‐like cells and, therefore, might be expected to severe the fibrosis. In the present study fibroblasts were, however, administered after the induction of fibrosis and were not exposed to activation stimuli and, therefore, probably remained inactivated. Furthermore, cells were injected during pHx which initiated liver regeneration. Altogether, this might explain why administration of fibroblasts did not lead to more severe fibrosis. The question remains how this local MSC treatment could reduce the collagen content in these regenerating livers. Hepatocyte differentiation of MSCs is one of the suggested working mechanisms of MSC therapy in literature.^15^, ^16^, ^39^ However, one could argue that MSC differentiation might affect the process of fibrogenesis. MSCs, which are differentiated into hepatocyte‐like cells, are known to improve liver function but are less able to affect the resolution of fibrogenesis. Therefore, we speculate that hepatocyte differentiation is not the driving mechanism for the observed collagen reduction in the present study.

Parekkadan et al proposed that a reduction in proliferation of stellate cells and silencing of myofibroblasts was because of cytokines (IL‐10, HGF, VEGF and IGF‐1) secreted by MSCs leading to less ECM production in the liver.^20^, ^40^, ^41^ Previous results from our group, using the same murine MSCs, also showed expression of these pro‐regenerative and anti‐fibrotic cytokines.^24^, ^42^ In literature, it was also suggested that the effect of MSC treatment depends on myofibroblast/MSC ratio, which might pose an explanation for the dose dependency.^20^ Altogether, it is highly suggestive that paracrine secretion of cytokines such as HGF and IGF‐1 by the MSCs directly target the process of fibrogenesis and might explain the observed collagen reduction. However, further in‐depth studies are needed to assess these suggested mechanisms.

Our study showed that different results between previous studies of MSC therapy might be explained by the use of different study designs. Variables like disease stage, MSC dosage, route of administration and even the local effect in the liver might explain these different and sometimes contradictive outcomes.^30^ For example, clinical studies mostly focus on systemic administration of MSCs. The present study showed that local MSC treatment had an on‐site therapeutic effect while iv treatment was ineffective. Because of this finding, one could speculate that the effect of MSCs in patients could be enlarged when MSCs are locally administered at multiple injection sites over the liver combined with a trigger for regeneration by a pHx comparable as to the treatment of perianal fistulas in Crohns disease.^43^ The set‐up of this observational study was to evaluate the effects of different study designs of MSC therapy on the reversal of fibrogenesis at the end of the regenerating process. In the present study, the most optimal MSC therapy was identified but owing to the observational nature of the study we did not assess the underlying working mechanisms. A follow‐up study where mice are sacrificed at multiple time‐points during the regeneration process is needed to unravel the underlying working mechanism of this novel MSC therapy. Possible effects on proliferation of endogenous liver cells need to be examined at an earlier time‐point, because in the present study all the livers are already fully regenerated. Furthermore, as portal infusion is comparable to local administration one might speculate that portal infusion also has a functional effect that might be considered for clinical use. However, this administration route was not tested because it was impossible to perform a portal infusion of MSCs in mice.

In conclusion, our data show that local administration of MSCs in combination with pHx enhances reduction of relative collagen content in regenerating livers. This observation might potentially lead in the future to an attractive novel treatment strategy of patients with liver fibrosis and cirrhosis.

## CONFLICT OF INTEREST

The authors confirm that there are no conflicts of interest.

## AUTHOR CONTRIBUTIONS

DH: analysis and interpretation of data, statistical analysis, drafting of the manuscript. DH, MCB, ESMJM, IM: acquisition of data. MCB, IM, LJACH: technical advice. MJC, BH, HWV: study concept and design. HWV: project leader. All authors critically reviewed the manuscript and approved the final submitted version.

## Data Availability

The data that support the findings of this study are available from the corresponding author upon reasonable request.
