# Supplementary material for: Local but not systemic administration of mesenchymal stromal cells ameliorates fibrogenesis in regenerating livers
Source: J Cell Mol Med. 2019 Jun 27;23(9):6238–50. doi: 10.1111/jcmm.14508 (PMC6714167; doi:10.1111/jcmm.14508)
Supplement: Supplementary file 1 [file JCMM-23-6238-s001.docx]

**Local but not systemic administration of mesenchymal stromal cells ameliorates fibrogenesis in regenerating livers**

**Supplementary information**

Danny van der Helm, Marieke C. Barnhoorn, Eveline S.M. de Jonge-Muller, Ilse Molendijk, Luuk J.A.C. Hawinkels, Minneke J. Coenraad, Bart van Hoek^#^, Hein W. Verspaget^#,^*

*Department of Gastroenterology and Hepatology, Leiden University Medical Center, Leiden, The Netherlands*

^#^ Joint senior authorship

***Corresponding Author:**

Prof. dr. H.W. Verspaget MSc, PhD

Department of Gastroenterology and Hepatology

Leiden University Medical Center

P.O. Box 9600, 2300 RC Leiden

The Netherlands

Telephone/Fax: +31 71 526 2680/+31 71 524 8115

E-mail: [H.W.Verspaget@lumc.nl](mailto:H.W.Verspaget@lumc.nl)

**
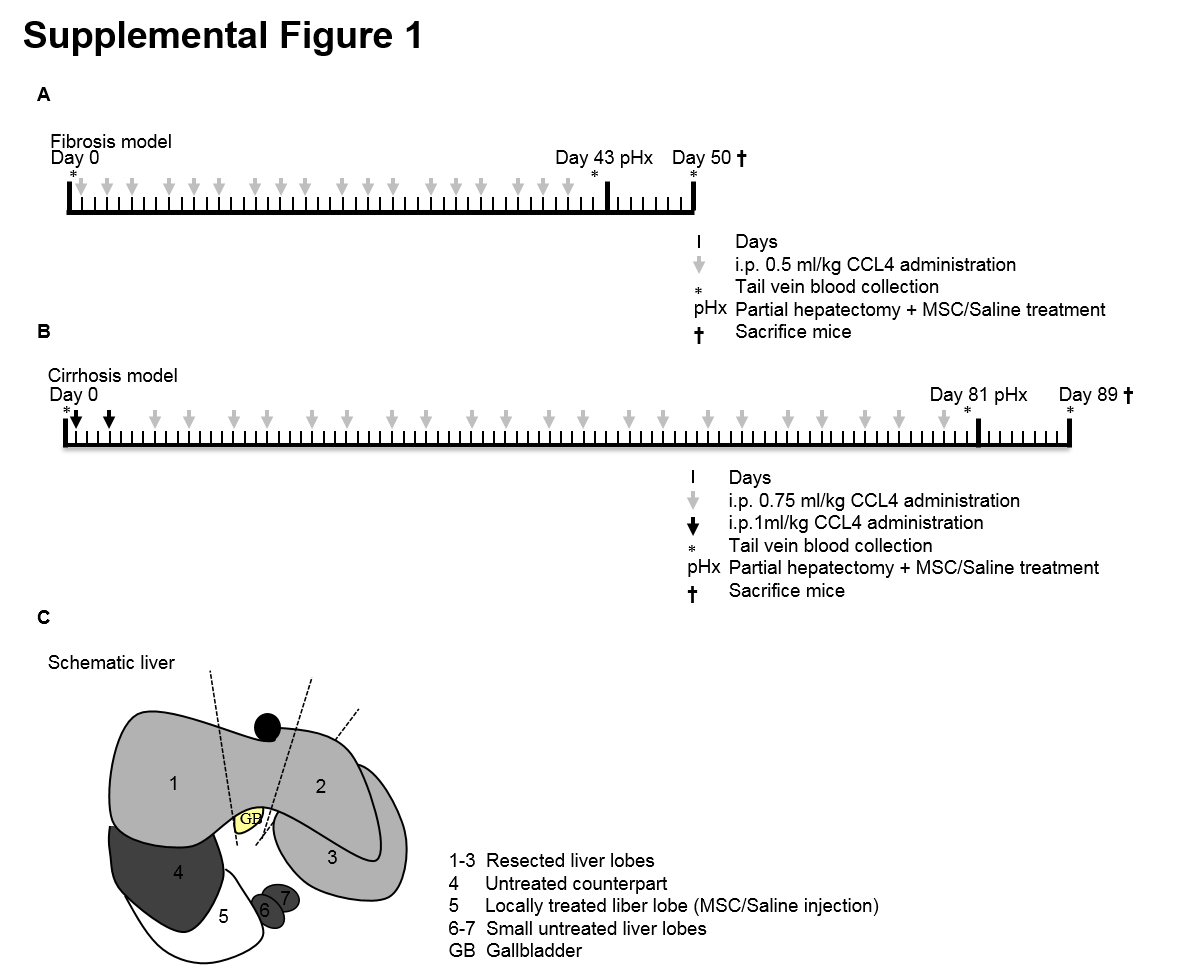
Supplementary Fig. 1. Induction of fibrotic and cirrhotic mouse models**

(A) Schematic overview of the induction of fibrosis. (B) Schematic overview of the induction of cirrhosis. (C) During partial hepatectomy, with concomitant local treatment, three lobes were resected (1-3). Lobe 4 was untreated and lobe 5 received vehicle, MSCs or fibroblasts.

**Supplementary Fig. 2. Lobuli closure scoring method**

Central Vein

Portal triad

Sirius-Red stained collagen septa

Septa

Healthy

0% closure

Fibrosis

70% closure

Cirrhosis

100% closure

Schematically overview and explanation of the lobuli closure score. (A) Typical healthy hexagonal liver structure (lobuli) consisting of 6 portal triads and 1 central vein. (B) Example of lobuli during fibrotic induction in which septa between the triads have begun to form (estimated closure is 70%). (C) Example of lobuli during cirrhotic induction in which bridging between the triads is observed (closure is 100%).

**Supplementary Fig. 3. MSC and fibroblast cell characterization**


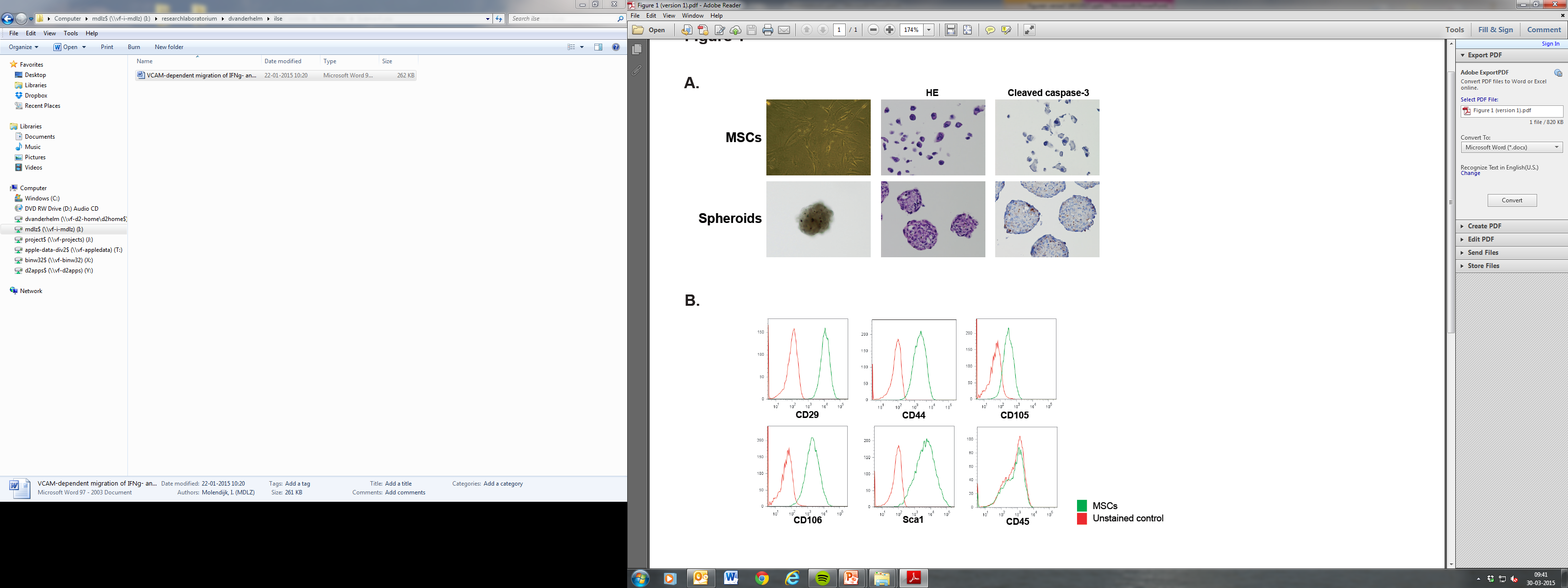

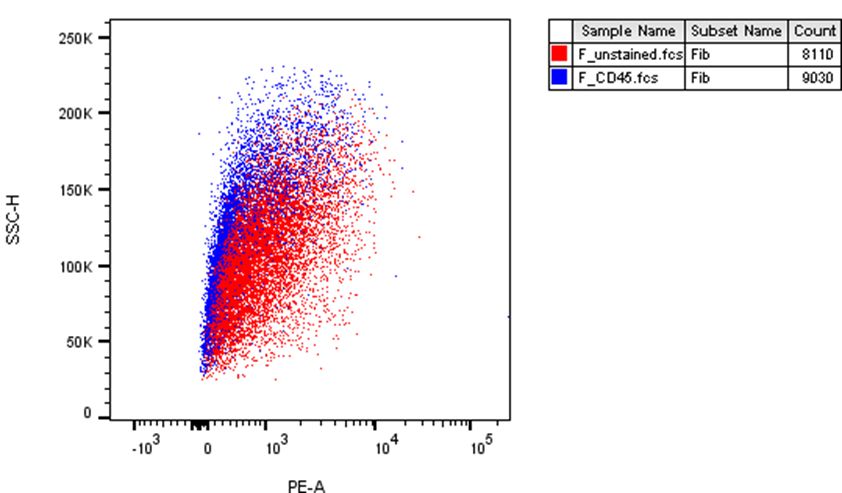

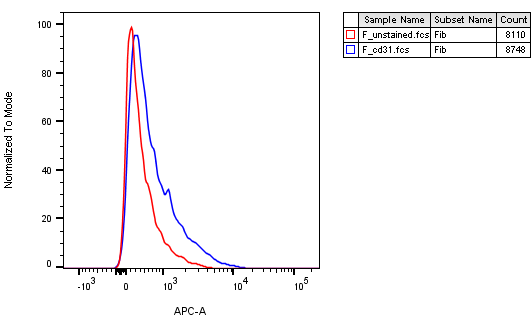

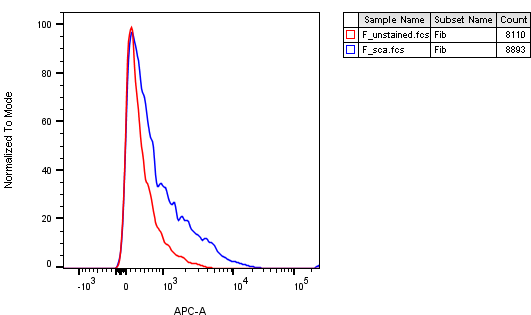

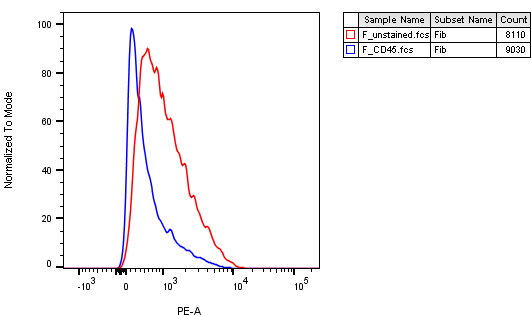

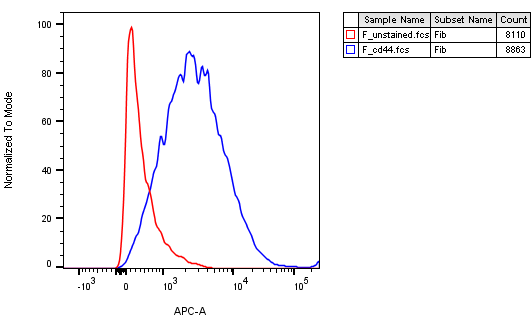

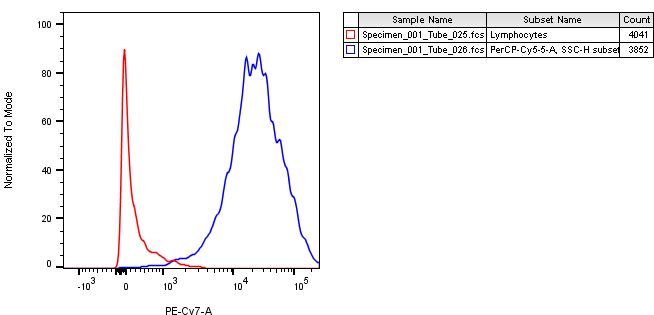

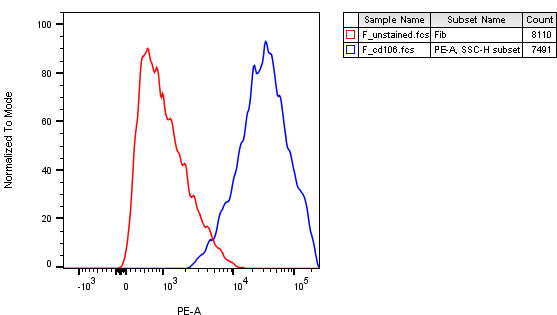

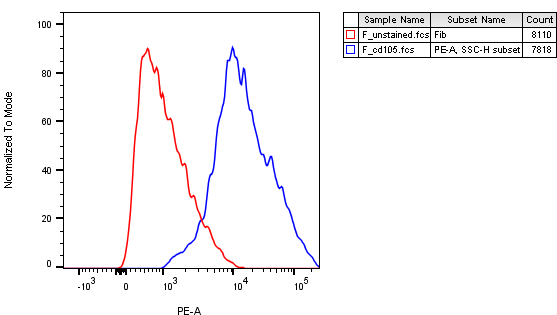


Unstained controls

Fibroblasts

**B**

CD29

CD44

CD105

CD106

SCA-1

CD45

CD31


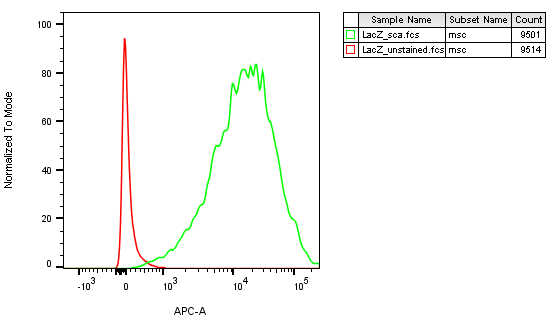

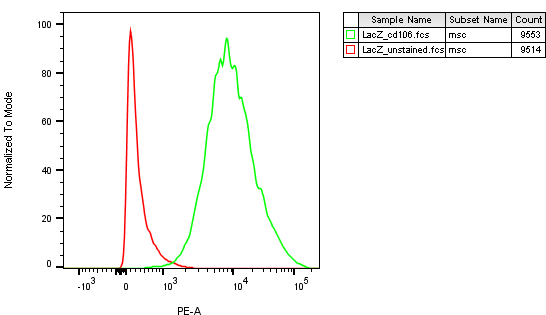

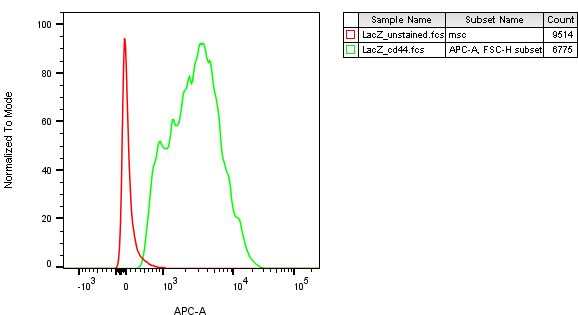

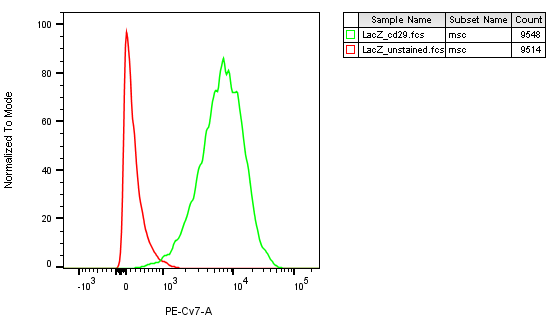

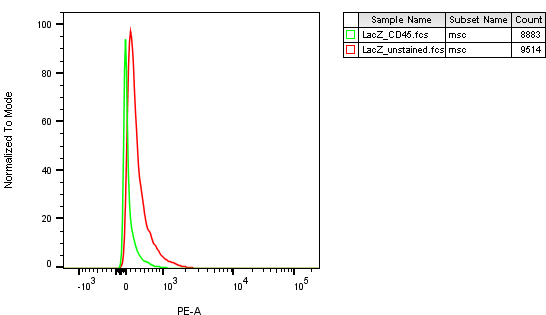


SCA-1

CD45

**A**


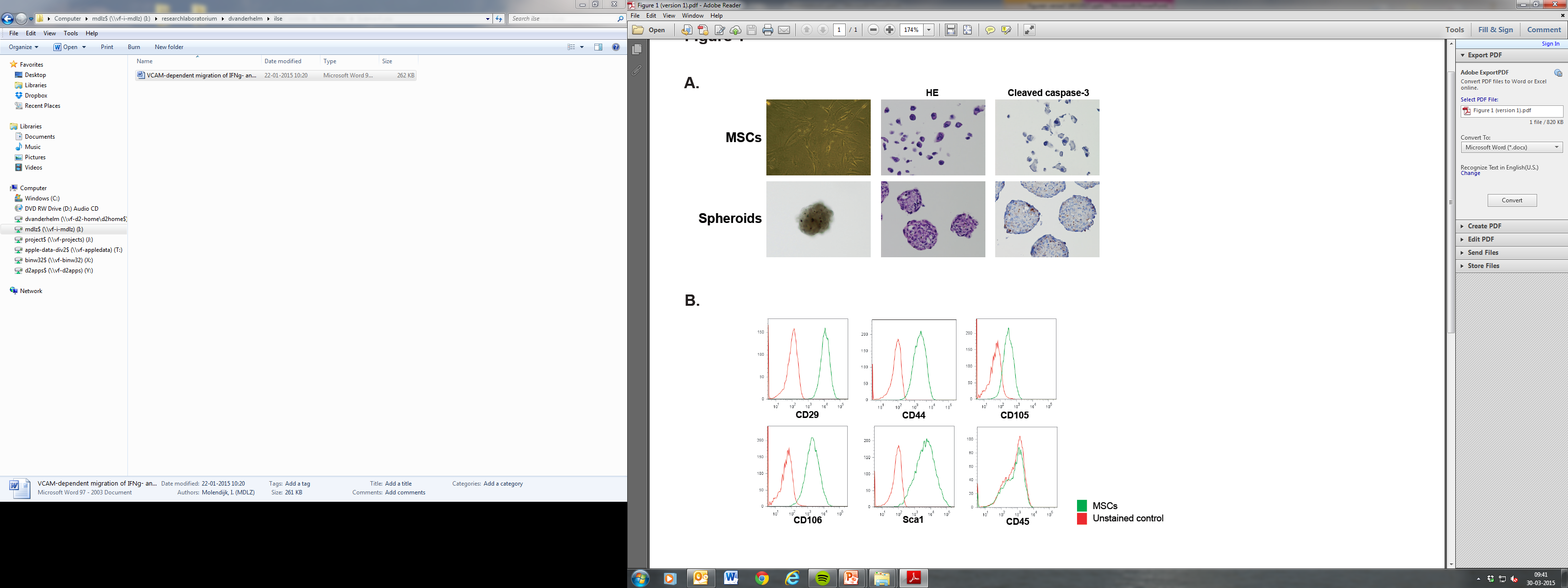

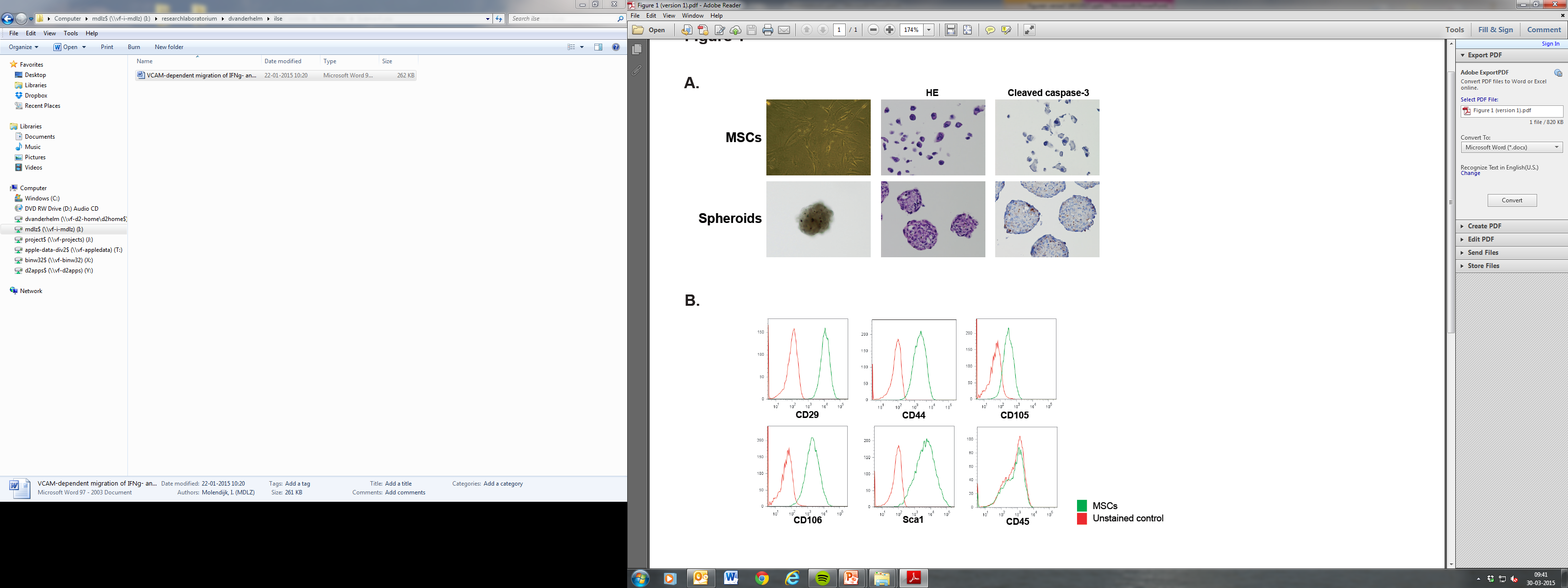


Unstained controls

MSCs


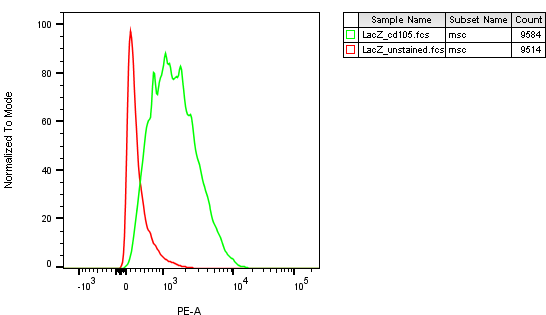


CD29

CD44

CD105

CD106


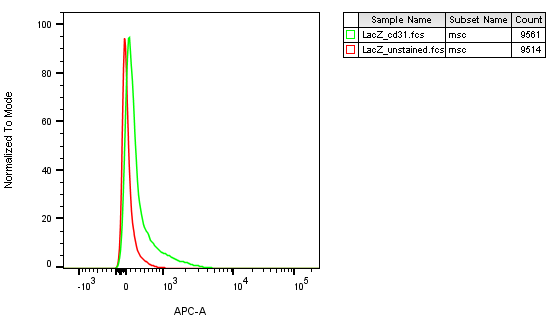


CD31

Bone marrow-derived and liver-derived fibroblast were isolated from 10 week old actin-GFP C57Bl/6Jico mice. (A) MSCs and (B) Fibroblasts were characterized by flow cytometry for the presence or absence of CD106, CD105, CD29, CD44, SCA-1, CD45 and CD31 membrane proteins.

Lipid droplets


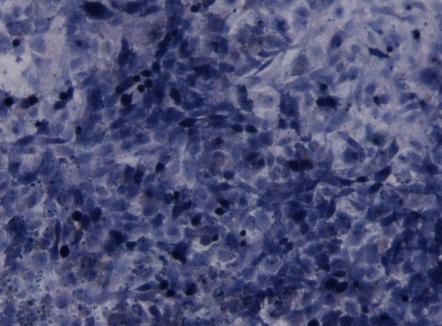

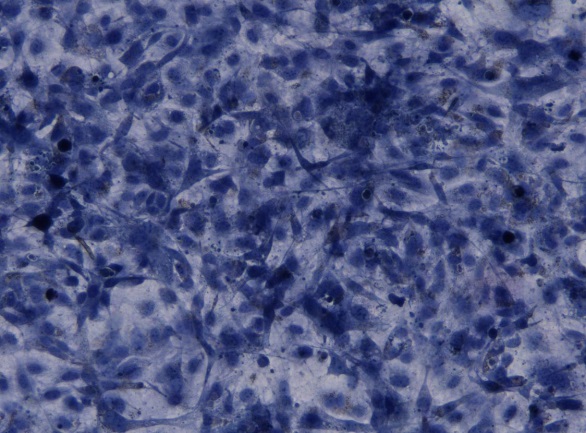

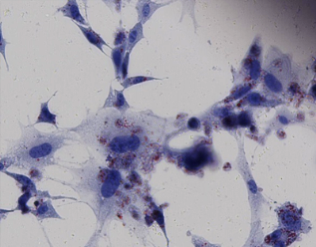

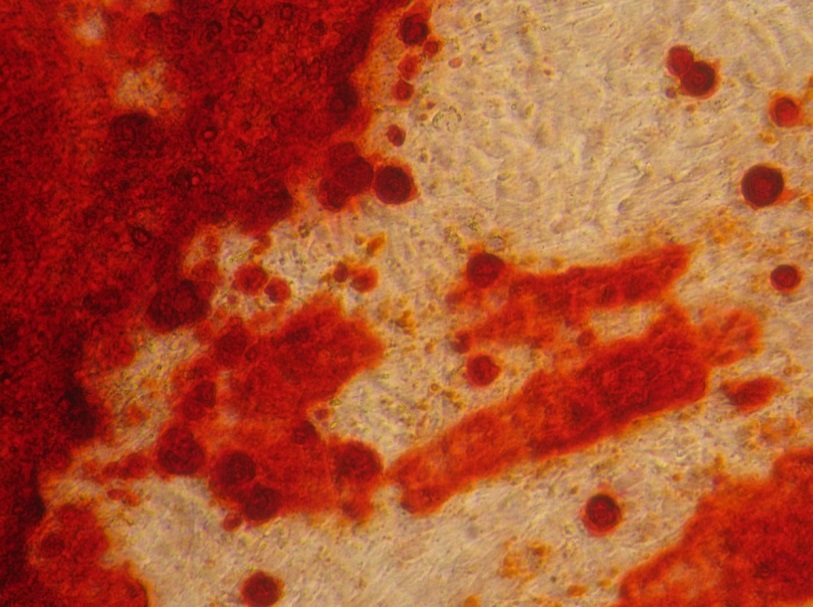

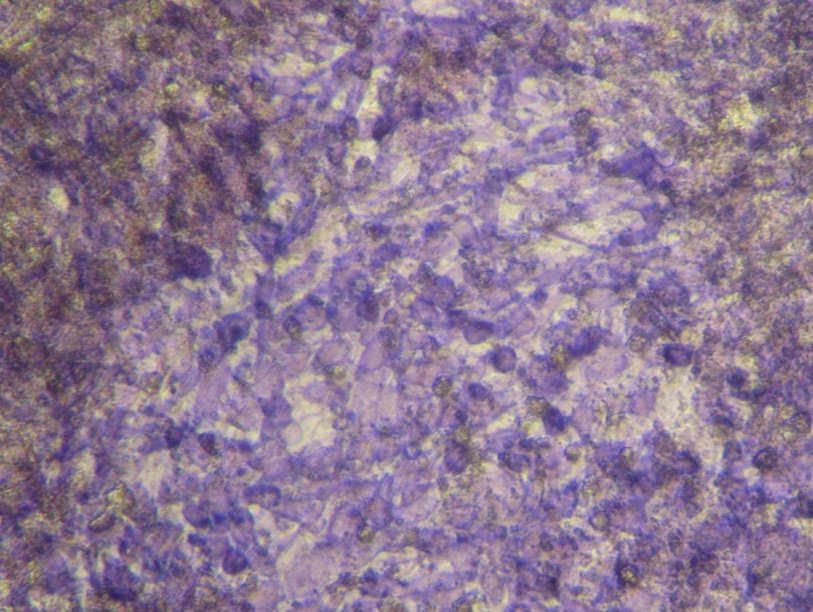


Alkaline phosphatase

Calcium

Lipid droplets


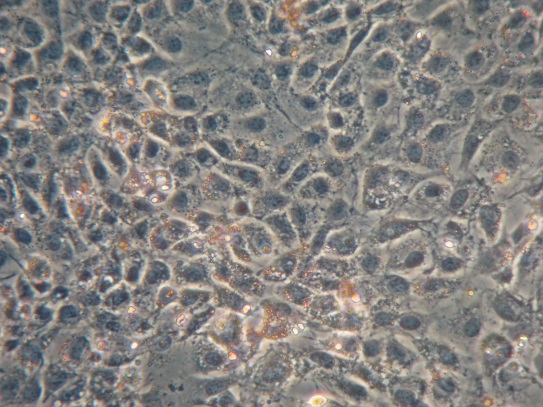


MSCs

Fibroblasts

**Supplementary Fig. 4. Osteoblast and adipocyte cell differentiation.**

MSCs and Fibroblasts were isolated from the liver of 10 week old actin-GFP C57Bl/6Jico mice and characterized by adipocyte differentiation visualized by cytoplasmic lipid droplets (oil-red-o) staining (black arrows) and osteoblast differentiation visualized by upregulation of alkaline phosphatase (fast blue staining) and calcium deposit (Alizarin red staining).

**A**

**B**

**C**

**D**

**Cirrhosis**

**Fibrosis**

**Supplementary Fig. 5. Aminotransferase levels after liver regeneration**

After CCL4 induced fibrosis and cirrhosis, mice underwent partial hepatectomy and were divided in three groups which received a local treatment of vehicle, 1x10^6^ or 2x10^6^ MSCs. Eight days after treatment blood from the tail vein blood was collected. (A,B) ALT and AST serum levels of treated fibrotic mice. (C,D) ALT and AST serum levels of treated cirrhotic mice.

**Supplementary material and methods**

**MSC and fibroblast characterisation**

Flow cytometry was used to characterise the isolated cells. The isolated cells were incubated for 30 minutes with fluorescent conjugated antibodies: CD29-PE-Cy7, C45-PE, SCA-1-APC, CD31-APC (eBioscience, Vienna, Austria), CD44-APC, CD105-PE or CD106-PE (BD Pharmingen, San Diego, CA, USA). Next, the fluorescence was measured by LSR II flow cytometer (BD Biosciences, San Diego, CA, USA), with FACS-diva software (version 8.7.1., Tree Star Inc. Ashland, OR, USA). Data analysis was performed with FlowJow software (version 8.7.1., Tree Star Inc. Ashland, OR, USA). Furthermore, the ability of MSCs and fibroblasts to differentiate in to osteoblasts and adipocytes was tested. In short, MSCs and fibroblasts were cultured for 21 days with osteogenic or adipogenic differentiation medium. Osteogenic differentiation medium consists of complete medium supplemented with 10nM dexamethason, 50μg/ml ascorbic acid and 10mM β-glycerophosphate (all from Sigma-Aldrich Chemie BV, Zwijndrecht, The Netherlands). Adipogenic differentiation medium consists of complete medium supplemented with 1µM dexamethason, 5µM insulin, 100μM indomethacin and 0.5mM 3-isobutyl-1-methylxanthine (all from Sigma-Aldrich Chemie BV, Zwijndrecht, The Netherlands). Osteogenic differentiation was verified by alkaline phosphatase expression and calcium deposition confirmed by fast blue and alizarin red staining respectively (both Sigma-Aldrich Chemie BV, Zwijndrecht, The Netherlands). Adipogenic differentiation was verified by the formation of lipid droplets with an oil-red-o staining (Sigma-Aldrich Chemie BV, Zwijndrecht, The Netherlands).
